# Performance of swab-based MiniDock MTB test for the diagnosis of pulmonary TB

**DOI:** 10.5588/ijtldopen.26.0272

**Published:** 2026-09-15

**Authors:** K.U. Eman, Y.L. Jackson, U.R. Lodhi, B. Kirubi, S. Qureshi, J. Creswell, S. Tahseen

**Affiliations:** 1 University of Geneva, Geneva, Switzerland;; 2 Dopasi Foundation, Islamabad, Pakistan;; 3 Geneva University Hospital and University of Geneva, Geneva, Switzerland;; 4 Stop TB Partnership, Geneva, Switzerland;; 5 Communicable Disease Control, Sindh, Pakistan.

**Keywords:** tuberculosis, Pakistan, diagnostic accuracy, NPOC-NAAT, sputum swab, tongue swab, pulmonary TB

Dear Editor,

The introduction of WHO-recommended rapid molecular diagnostics (WRD) beginning with Xpert MTB/RIF in 2010 improved the speed and accuracy of TB diagnosis and rifampicin resistance detection. However, 14 years later, only 54% of notified TB patients were initially tested using WRD, indicating persistent gaps in coverage.^1^ Key challenges include limited access to WRD at primary health care levels,^2^ largely due to infrastructure and cost constraints, as well as continued reliance on sputum-based specimens.^3,4^ Lower cost assays requiring minimal infrastructure and training are needed to improve access to molecular diagnostics. With investments from COVID-19 assay development, the TB diagnostic pipeline has expanded considerably,^5^ including low cost, near point-of-care (NPOC) nucleic acid amplification tests aligning with WHO target product profiles.^1,6^ One of these tests, MiniDock MTB (Guangzhou Pluslife Biotech Co. Ltd., China) was recommended by WHO in March 2026 to replace smear microscopy for adults and adolescents with presumptive pulmonary TB, and supports tongue swab–based testing when sputum cannot be obtained.^7^ To date, only three studies on diagnostic performance have been published,^8–10^ two multicentre studies based in health facility settings,^8,9^ and an evaluation from Cameroon that also included community-recruited participants and self-collected swabs.^10^ Performance of diagnostic tests will vary by the clinical setting and disease spectrum, and more studies in different settings are key to better understanding test performance.^11,12^

We conducted a prospective cross-sectional diagnostic accuracy study to evaluate MiniDock MTB for sputum and tongue swabs for TB diagnosis under routine programmatic conditions. The study was performed at the chest clinic of a tertiary care hospital in Karachi, Pakistan, prior to WHO guidance, as part of new tools evaluation in Pakistan. Adults and adolescents (≥10 years) with signs and symptoms of pulmonary TB referred to the TB laboratory were consecutively enrolled between 15 November 2025 and 29 January 2026 after informed consent. Trained staff collected brief demographic and clinical data, obtained a tongue swab, and instructed participants to provide two spontaneously expectorated sputum specimens. One sputum specimen was tested locally using Xpert MTB/RIF Ultra (Ultra) (Cepheid, Sunnyvale, CA, USA), and a sputum swab was prepared from the same specimen. Both swab specimens were tested using MiniDock MTB in accordance with the manufacturer’s instructions, and results were recorded based on the binary detection output. The second sputum specimen was transported (same day) to the Provincial Reference Laboratory, Sindh (same city), and processed for culture using the Mycobacterial Growth Indicator Tube (MGIT) system (Becton Dickinson, Franklin Lakes, NJ, USA), and a smear was prepared from sediment and stained with auramine for acid-fast bacilli (AFB) microscopy (Supplementary Data Figure S1). MiniDock MTB performance on sputum and tongue swabs was assessed against MGIT culture. Sensitivity and specificity with 95% confidence intervals were calculated. Sputum swab performance was assessed in subgroups. Paired sensitivity differences were assessed using McNemar’s test. Analyses were conducted in Stata 13. The study was approved by the National Bioethics Committee, Islamabad, Pakistan (4-87/NBCR-1231/24-25/1898).

Overall, 323 participants were enrolled, including 152 women (47.1%). The median age was 40 years (interquartile range 25–55; range 12–106), with similar distribution between women and men. All participants were symptomatic: cough (99%), fever (78%), weight loss (61%), and night sweats (36%). Previous TB treatment was reported in 93 (29%), and none were known to be HIV-positive. Tongue swabs were collected from all 323 participants; 320 (99.1%) were able to expectorate sputum, and 318 provided two specimens of adequate volume for all tests. MiniDock MTB positivity rates were 15.8% (51/322) for tongue swab (one invalid excluded), 20.4% (65/318) for sputum swab (two invalid excluded), and 14.5% (46/318) for AFB microscopy (Supplementary Data Table S1). All three individuals unable to produce sputum tested tongue swab negative on MiniDock MTB (Supplementary Data Figure S1).

Among 318 participants tested with Ultra, 63 (19.9%) were positive (one invalid excluded). Semi-quantitative results were high in 34.9% (22/63), medium in 23.8% (15/63), low in 20.6% (13/63), very low in 12.7% (8/63), and trace in 7.9% (5/63) – see Supplementary Data Table S1. Culture was performed for 318 participants; after excluding 33 contaminated cultures, positivity was 22.5% (64/285) – see Supplementary Data Table S1. Culture positivity among Ultra-positive participants did not differ by history of previous TB treatment (88.1% vs. 88.9%; Fisher’s exact *P* = 1.00). Altogether, 83 specimens were positive on at least one test, of which 17 were positive on a single test only: tongue swab (n = 3), sputum swab (n = 3), Ultra (n = 2), and culture (n = 9). Overlap of positive results across four diagnostic tests is shown in Supplementary Data Figure S2.

MiniDock MTB performance is summarised in the Table. Against culture, sputum swab sensitivity was comparable to Ultra (*P* = 1.00) and higher than microscopy (*P* = 0.02) with sensitivity (81.3% vs. 85.4%) and specificity (96.8% vs. 97.6%) aligning with WHO-pooled estimates.^7^ Subgroup analyses showed no significant variation across most demographic and clinical characteristics although sensitivity was significantly higher among smear-positive and Ultra-positive participants compared to their negative counterparts (*P* < 0.001) – see Supplementary Data Table S2. In contrast, tongue swab performance was lower than WHO-pooled estimates, with sensitivity comparable to microscopy (*P* = 1.00) but lower than Ultra (*P* = 0.0063). Variability in the performance of tongue swab has also been reported for Ultra^13^ and MiniDock MTB across settings,^8,9^ and reduced sensitivity is observed among individuals with very low or trace results (Supplementary Data Table S1).^10,14^ The comparatively lower performance of tongue swab–based testing observed in our study likely reflects a higher proportion of paucibacillary TB cases. Among 73 participants positive by Ultra and/or culture, 26 had low, very low, or trace Ultra results and 10 were culture-positive/Ultra negative. Additionally, challenges in consistently adhering to recommended pre-collection restrictions (e.g., avoiding eating, drinking, or tooth brushing within 30 min before collection)^15^ under routine programmatic conditions may have resulted in suboptimal swab collection in some, with a reduced amount of mycobacterial material available for detection, potentially contributing to lower sensitivity.

**Table.** Diagnostic accuracy of near point-of-care MiniDock MTB using sputum and tongue swabs, and comparator tests, against mycobacterial culture and Xpert MTB/RIF Ultra in a tertiary care setting in Pakistan.

Diagnostic test	N	TP	FP	FN	TN	Sensitivity	Specificity	PPV	NPV
						(95% CI)	(95% CI)	(95% CI)	(95% CI)
A. Against mycobacterial culture									
MiniDock MTB sputum swab	285	52	7	12	214	81.3%	96.8%	88.1%	94.7%
						(69.5–89.9)	(93.6–98.7)	(77.1–95.1)	(90.9–97.2)
MiniDock MTB	284	42	6	21	215	66.7%	97.3%	87.5%	91.1%
Tongue swab						(53.7–78.0)	(94.2–99.0)	(74.8–95.3)	(86.7–94.4)
AFB smear	285	44	0	20	221	68.8%	100.0%	100.0%	91.7%
						(55.9–79.8)	(98.3–100)	(92.0–100)	(87.5–94.9)
MTB/RIF-Ultra including trace	282	53	7	10	212	84.1%	96.8%	88.3%	95.5%
						(72.7–92.1)	(93.5–98.7)	(77.4–95.2)	(91.9–97.8)
MTB/RIF-Ultra excluding trace	277	52	3	10	212	83.9%	98.6%	94.5%	95.5%
						(72.3–92.0)	(96.0–99.0)	(84.9–98.9)	(91.9–97.8)
B. Against Xpert MTB/RIF Ultra (including trace)									
MiniDock MTB	315	58	7	5	245	92.1%	97.2%	89.2%	98.0%
Sputum swab						(82.4–97.4)	(94.4–98.9)	(79.1–95.6)	(95.4–99.3)
MiniDock MTB	316	45	6	17	248	72.6%	97.6%	88.2%	93.6%
Tongue swab						(59.8–83.8)	(94.9–99.1)	(76.1–95.6)	(89.9–96.2)
AFB smear	315	45	1	18	251	71.4%	99.6%	97.8%	93.3%
						(58.7–82.1)	(97.8–100)	(88.5–99.9)	(89.6–96.0)
C. Against Xpert MTB/RIF Ultra (excluding trace)									
MiniDock MTB	310	56	7	2	245	96.6%	97.2%	88.9%	99.2%
Sputum swab						(88.1–99.6)	(94.9–98.9)	(78.4–95.4)	(97.1–99.9)
MiniDock MTB	311	44	6	13	248	77.2%	97.6%	88.0%	95.0%
Tongue swab						(64.2–87.3)	(94.9–99.1)	(75.7–95.5)	(91.6–97.3)
AFB smear	310	44	1	14	251	75.9%	99.6%	97.8%	94.7%
						(62.8–86.1)	(97.8–100)	(88.2–99.9)	(91.3–97.1)

AFB = acid-fast bacilli; N = number of participants with valid results on culture and index or comparator test; TP = true positive; FP = false positive; FN = false negative; TN = true negative; PPV = positive predictive value; NPV = negative predictive value; CI = confidence interval.

Our findings reinforce WHO recommendations that sputum should remain the preferred specimen for TB diagnosis, as tongue swab testing provided no added value in our study conducted in a clinical setting, with most of the individuals being symptomatic, HIV-negative, and able to expectorate. However, if tongue swab testing performance in individuals with presumptive TB unable to produce sputum is similar to that observed in the study group, it could still make a meaningful contribution to microbiological TB detection in peripheral and community settings where no alternative diagnostic option is available. Diagnostic performance may improve with optimised collection approaches, including self-collection strategies, and refinement of assay protocols.^3^ Low invalid rates across both specimen types support the operational feasibility of the NPOC platform in decentralised settings.

A key strength of our study is evaluation under routine programmatic conditions. Limitations include potential specimen-related discordance due to use of different samples across tests, and possible overestimation of AFB microscopy performance due to use of concentrated smear and expert reading. The single-centre design and inclusion of only symptomatic and HIV-negative individuals able to expectorate limit generalisability.

In conclusion, MiniDock MTB on sputum swab demonstrated performance comparable to Ultra and has potential to expand access to molecular TB diagnosis in decentralised settings. Tongue swab–based testing offers a non-invasive option for individuals unable to expectorate; however, it should not replace sputum-based diagnostics. More studies are needed across a variety of settings to better understand the best use for TB programmes as performance may vary by disease spectrum.

## Supplementary Material
