# Supplementary material for: Performance of swab-based MiniDock MTB test for the diagnosis of pulmonary TB
Source: IJTLD Open. 2026 Sep 15;3(9):604–6. doi: 10.5588/ijtldopen.26.0272 (PMC13577207; doi:10.5588/ijtldopen.26.0272)
Supplement: Supplementary file 1 [file ijtldopen26-0272_supplementarydata1.pdf]

Supplementary Data

Figure-S1: Flow diagram showing participant recruitment and diagnostic cascade

Table-S1: Distribution of culture, MiniDock MTB sputum swab, tongue swab, and AFB smear results stratified by Xpert MTB/RIF Ultra semi-quantitative categories.

Figure-S2. Venn diagram showing overlap of tuberculosis-positive results across four diagnostic tests. (n=74)

Table S2: Subgroup analysis of the diagnostic performance of the Minidock MTB sputum swab against culture

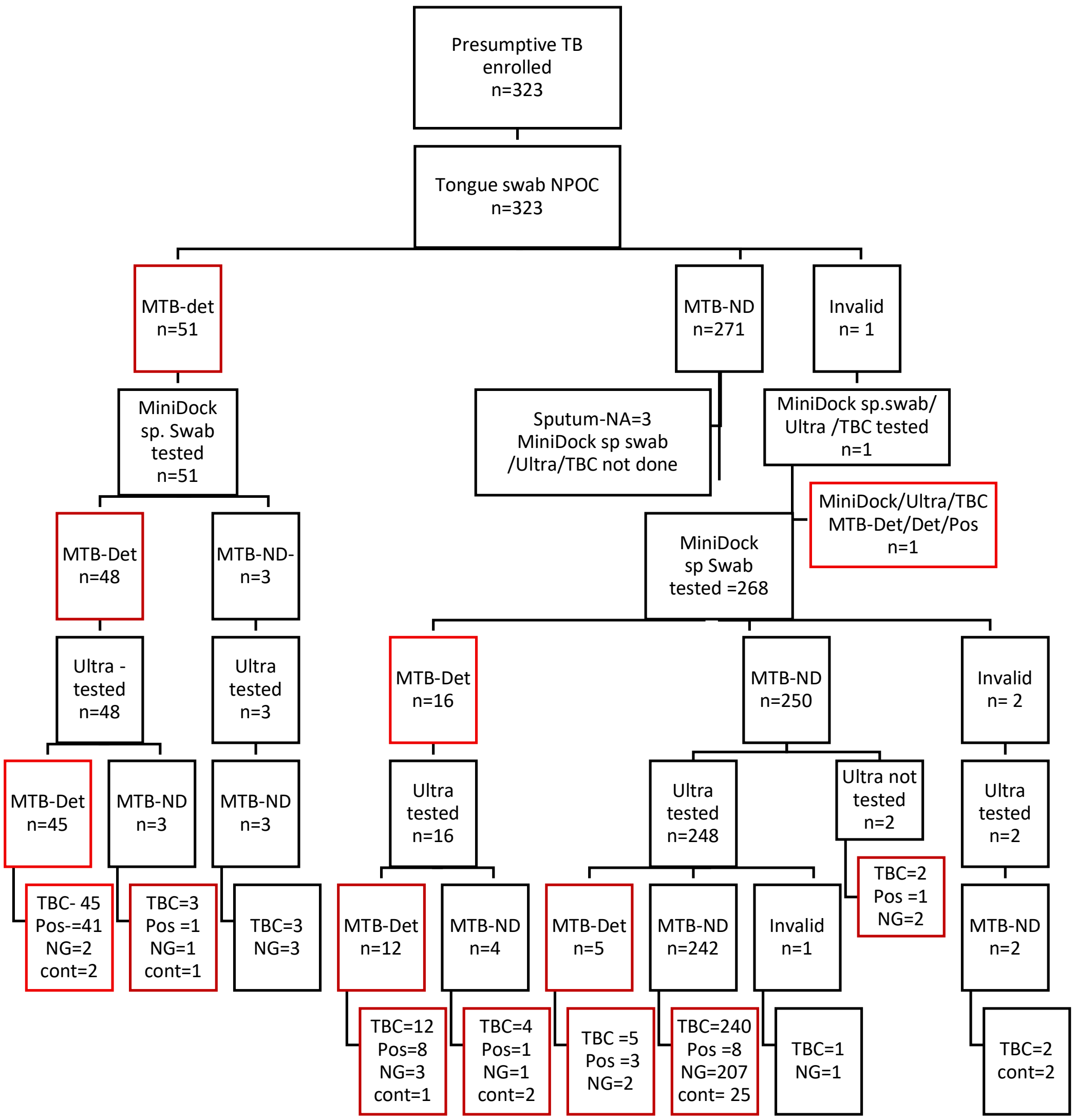

Figure-S1: Flow diagram showing participant recruitment and diagnostic cascade

Sp Swab =Sputum swab, Ultra = Xpert MTB/RIF Ultra; TBC=TB culture, MTB = Mycobacterium tuberculosis; Pos = positive; Neg = negative; Det = detected; ND = not detected; NG = no growth; cont = contaminated.

Positive results are indicated by red boxes.

Table-S1: Distribution of culture, MiniDock MTB sputum swab, tongue swab, and AFB smear results stratified by Xpert MTB/RIF Ultra semi-quantitative categories.

Xpert MTB/RIF ULTRA		Culture			Sputum swab			Tongue swab			AFB smear		
		**Pos**	**Neg**	**NA**	**Pos**	**Neg**	**NA**	**Pos**	**Neg**	**NA**	**Pos**	**Neg**	**NA**
MTB-High	22	21	0	1*	22	0	0	22	0	0	22	0	0
MTB-Medium	15	14	0	1*	15	0	0	13	1	1	14	1	0
MTB-Low	13	11	2	0	12	1	0	8	5	0	8	5	0
MTB-Very Low	8	6	1	1*	7	1	0	1	7	0	0	8	0
MTB-Trace	5	1	4	0	2	3	0	1	4	0	1	4	0
**MTB-Pos (all)**	**63**	**53**	**7**	**3***	**58**	**5**	**0**	**45**	**17**	**1$^{\infty}$**	**45**	**18**	**0**
**MTB-Neg**	**254**	**10**	**212**	**30*+2****	**7**	**245**	**2$^{\infty}$**	**6**	**248**	**0**	**1**	**251**	**2****
Results invalid	1	0	1	0	0	1	0	0	1	0	0	1	0
Not done	5	1	1	3**	0	2	3**	0	5	0	0	2	3**
**Total**	**323**	**64**	**221**	**38**	**65**	**253**	**5**	**51**	**271**	**1**	**46**	**272**	**5**

Pos = positive; Neg = negative; NA = not available; MTB = *Mycobacterium tuberculosis*; AFB = acid-fast bacilli; * contaminated (n = 3+30), ** not done $^{\infty}$ invalid

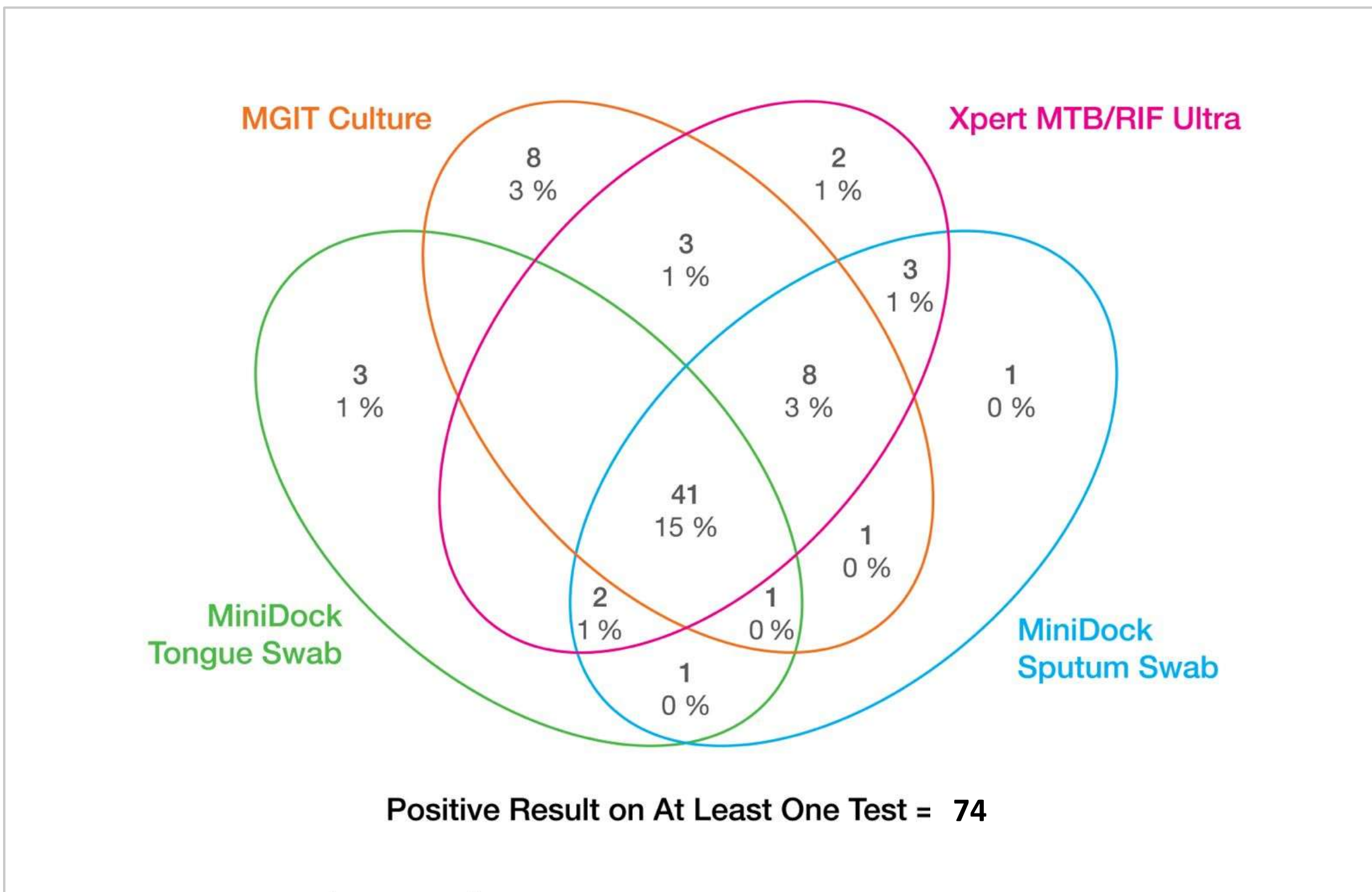

Figure-S2. Venn diagram showing overlap of tuberculosis-positive results across four diagnostic tests. (n=74)

Only participants (n=281) with interpretable results for all four diagnostic methods (tongue swab, sputum swab, Xpert Ultra, and culture) were included in the analysis

Table S2: Subgroup analysis of the diagnostic performance of the Minidock MTB sputum swab against culture

**Subgroups**		All enrolled	**N***	**TP**	**FN**	**FP**	**TN**	**Sensitivity (95%CI)**	**Specificity (95%CI)**
Gender	Female	152	137	22	5	2	108	81.5% (61.9- 93.7)	98.2% (93.6- 99.8)
	Male	171	148	30	7	5	106	81.1% (64.8- 92.0)	95.5% (89.8- 98.5)
Age group	10-19yrs	36	30	7	2	1	20	77.8% (40.0 - 97.2)	95.2% (76.2- 99.9)
	20-29yrs	77	75	17	3	2	53	85.0% (62.1-96.8)	96.4% (87.5- 99.6)
	30-39yrs	42	32	10	0	0	22	100.0% (69.2- 100.0)	100.0% (84.6- 100.0)
	40-49yrs	54	46	5	3	0	38	62.5% (24.5 -91.5)	100.0% (90.7- 100.0)
	50-59yrs	56	51	7	2	4	38	77.8% (40.0 - 97.2)	90.5% (77.4 - 97.3)
	≥60yrs	58	51	6	2	0	43	75.0% (34.9 - 96.8)	100.0% (91.8 - 100.0)
TB symptoms	One	32	27	2	2	0	23	50.0% (6.8 - 93.2)	100.0% (85.2 - 100.0)
	Two	89	74	8	4	0	62	66.7% (34.9 - 90.1)	100.0% (94.2 - 100.0)
	Three	131	118	26	5	7	80	83.9% (66.3 - 94.5)	92.0% (84.1 - 96.7)
	Four	71	66	16	1	0	49	94.1% (71.3 - 99.9)	100.0% (92.7 - 100.0)
Previous history of TB	Yes	93	86	16	2	1	67	88.9% (65.3 - 98.6)	98.5% (92.1 - 100.0)
	No	230	199	36	10	6	147	78.3% (63.6 - 89.1)	96.1% (91.7 - 98.5)
AFB microscopy	Negative	272	241	9	11	7	214	45.0% (23.1- 68.5)	96.8% (93.6 - 98.7)
	Positive	46	44	43	1			97.7% (88.0-99.9)	
Xpert MTB/RIF Ultra	MTB-Not detected	254	222	2	8	2	210	20.0% (2.5 - 55.6)	99.1% (96.6 - 99.9)
	MTB-Detected	63	60	50	3	5	2	94.3% (84.3 - 98.8)	28.6%

N=Number of participants with valid results on culture and index test.

TP = true positive; FP = false positive; FN = false negative; TN = true negative.
